# Supplementary material for: To what extent is telehealth reported to be incorporated into undergraduate and postgraduate allied health curricula: A scoping review
Source: PLoS One. 2021 Aug 19;16(8):e0256425. doi: 10.1371/journal.pone.0256425 (PMC8376028; doi:10.1371/journal.pone.0256425)
Supplement: S1 Table — (PDF) [file pone.0256425.s002.pdf]

*S1 Table: Medline Search Strategy*

| |
|---|
| **1.** Allied Health Personnel/<br>**2.** Allied Health Occupations/<br>**3.** Occupational therapy/<br>**4.** Speech language pathology/<br>**5.** Physical Therapy Modalities/<br>**6.** Students/<br>**7.** Audiologist/<br>**8.** Chiropractic/<br>**9.** Nutritionists/<br>**10.** Orthoptics/<br>**11.** Osteopathic Physicians/<br>**12.** Podiatry/<br>**13.** Social Work/<br>**14.** Exercise Physiology/<br>**15.** Psychology, Educational/<br>**16.** Students, Health Occupations/<br>**17.** (under?graduate* or undergraduate* or postgraduate* or post?graduate* or master* or student* or allied health person* or allied health profession* or allied health occupat* or occupational therap* or physical therap* or speech?language pathology or audiolog* or chiropract* or diabetes educat* or educator, diabetes or nutritionist* or dietician* or dietitian* or exercise physiolog* or orthoptic* or pleoptic* or osteopath* or podiatr* or social work* or service* social or social service* or work, social or psychology, educational or under?graduate student* or undergraduate student* or post?graduate student* or postgraduate student* or college student* or health student* or university student* or graduate student* or social work student* or allied health person* or physical therapy modalities or osteopathic physician* or osteopathic medicine or psycholog* or students, health occupation* or audiology student* or chiropractic student* or chiropractic education* or podiatry education* or allied health education* or nutrition education or psychoeducation or psychology education or educational psychology or graduate psychology education or allied health occupation* or speech therapy or physical therap* or college student* or postgraduate student* or graduate student*). |
| **18.** 1 or 2 or 3 or 4 or 5 or 6 or 7 or 8 or 9 or 10 or 11 or 12 or 13 or 14 or 15 or 16 or 17 |
| **19.** Telemedicine/<br>**20.** Telerehabilitation/<br>**21.** (mobile health* or tele?health* or telehealth* or tele?medicine* or telemedicine* or e?health* or ehealth* or m?health* or mhealth* or remote rehabilitation* or virtual rehabilitation* or tele?rehabilitation* or telerehabilitation* or e?rehabilitation or tele?OT or teleOT or tele?occupational therap* or teleoccupational therap* or tele?speech path* or telespeech path* or tele?phys* or telephys* tele?audiolog* or teleaudiolog* or tele?chiro* or telechiro* or tele?nutrition* or telenutrition* or tele?diet* or telediet* or tele?orthopt* or teleorthopt* or tele?osteopathic phys* or teleosteopathic phys* or tele?podiat* or telepodiat* or tele?psycholog* or telepsycholog* or tele?social work* or telesocial work or e?occupational therap* or e?OT or e?speech path* or e?phys* or e?audiolog* or e?chiro* or e?chiro* or e?diet* or e?nutrition* or e?orthop* or e?osteopath* or e?podiat* or e?psycholog* or e?social work* or m?occupational therap* or m?OT or m?speech path* or m?phys* or m?audiolog* or m?chiro* or m?diet* or m?nutrition* or m?orthop* or m?osteopath* or m?podiat* or m?psycholog* or m?social work*). |
| **22.** 19 or 20 or 21 |
| **23.** Health Education/<br>**24.** exp Curriculum/<br>**25.** Program Development/<br>**26.** Health knowledge, attitudes, practice/ |

| | |
|---|---|
| **27.** *Universities/*<br>**28.** (health educat* or curriculum* or program develop* or syllabus* or course* or universit* or curriculum development or college* or educational program planning or educational programs or higher education or health knowledge, attitudes, practice). |
| **29.** 23 or 24 or 25 or 26 or 27 or 28 |
| **30.** 18 and 22 and 29 |
