# Supplementary material for: To what extent is telehealth reported to be incorporated into undergraduate and postgraduate allied health curricula: A scoping review
Source: PLoS One. 2021 Aug 19;16(8):e0256425. doi: 10.1371/journal.pone.0256425 (PMC8376028; doi:10.1371/journal.pone.0256425)
Supplement: Attachment — Submitted filename: Rebuttal letter 270721.docx [file pone.0256425.s003.docx]

**To what extent is telehealth reported to be incorporated into undergraduate and postgraduate allied health curricula: A scoping review**

Editor’s comment:

The manuscript is interesting and the review process is comprehensive and well described. The only major limitation is the rapid changes in telehealth and in online medical and health professions education brought about by the COVID-19 pandemic. The authors can include more recent studies conducted in this area as suggested by the reviewers. They can try to expand the search duration till 30th April 2021. This will provide a contemporary picture about this important area in medical and health professions education.

**Authors’ response**

Thank you for your comments and recommendations.
The systematic search has been updated as suggested with the search duration expanded to April 30^th^ 2021. As a result two further studies published in 2020 have been included.

**Comments to the Author**

1. Is the manuscript technically sound, and do the data support the conclusions?

The manuscript must describe a technically sound piece of scientific research with data that supports the conclusions. Experiments must have been conducted rigorously, with appropriate controls, replication, and sample sizes. The conclusions must be drawn appropriately based on the data presented.

Reviewer #1: Yes

Reviewer #2: Yes

**Authors’ response**
Thank you

2. Has the statistical analysis been performed appropriately and rigorously?

Reviewer #1: N/A

Reviewer #2: Yes

**Authors’ response**
Thank you

3. Have the authors made all data underlying the findings in their manuscript fully available?

The [PLOS Data policy](https://protect-au.mimecast.com/s/11BeC3QN2KT8m32WUQ3q_S?domain=plosone.org) requires authors to make all data underlying the findings described in their manuscript fully available without restriction, with rare exception (please refer to the Data Availability Statement in the manuscript PDF file). The data should be provided as part of the manuscript or its supporting information, or deposited to a public repository. For example, in addition to summary statistics, the data points behind means, medians and variance measures should be available. If there are restrictions on publicly sharing data—e.g. participant privacy or use of data from a third party—those must be specified.

Reviewer #1: Yes

Reviewer #2: Yes

**Authors’ response**
Thank you

4. Is the manuscript presented in an intelligible fashion and written in standard English?

PLOS ONE does not copyedit accepted manuscripts, so the language in submitted articles must be clear, correct, and unambiguous. Any typographical or grammatical errors should be corrected at revision, so please note any specific errors here.

Reviewer #1: Yes

Reviewer #2: Yes

**Authors’ response**
Thank you

5. Review Comments to the Author

Please use the space provided to explain your answers to the questions above. You may also include additional comments for the author, including concerns about dual publication, research ethics, or publication ethics. (Please upload your review as an attachment if it exceeds 20,000 characters)

**Reviewer #1:** While the paper is a well written article on a useful topic, the period chosen for the scoping review has become outdated because of the digital disruption caused by the COVID-19 pandemic. It may be appropriately revised with references to newer publications like:
1. [https://www.ncbi.nlm.nih.gov/pmc/articles/PMC7301824/](https://protect-au.mimecast.com/s/Wg4lC4QORYTjJPlRcMKDtt?domain=ncbi.nlm.nih.gov)
2. [https://journals.healio.com/doi/10.3928/01484834-20200921-06](https://protect-au.mimecast.com/s/ei-_C5QP91Tn086wTNCIVC?domain=journals.healio.com)
3. [https://www.sciencedirect.com/science/article/abs/pii/S1555415520307261](https://protect-au.mimecast.com/s/jAXpC6XQRzU3okyZu20h2S?domain=sciencedirect.com)

**Authors’ response**
Thank you for the comment. In response, the scoping review has been updated to cover the period to April 30^th^, 2021.

Thank you also for the suggested additional articles.

Of these, No 2 by Rutledge et al was also found in the updated search and met the inclusion criteria, and has been inclusion in the scoping review.

24. Rutledge C, Hawkins EJ, Bordelon M, Gustin TS. Telehealth Education: An Interprofessional Online Immersion Experience in Response to COVID-19. J Nurs Educ. 2020 Oct 1;59(10):570-576. doi: 10.3928/01484834-20200921-06. PMID: 33002163.

The other two suggested articles did not meet the eligibility criteria. No 1 was not the design for inclusion and No 3 in nurse practitioners was not in the allied health population group to be included in this review.

**Reviewer #2**: Kindly check the sentence in lines 39 and 40 for correctness.
The current review is performed methodically and reported well, considering that there are no concerns about dual publication and the study is conducted with sound research ethics in place.

**Authors’ response**
Thank you for the comment.
The sentence in lines 39 and 40 have been checked for correctness.

Previously the Results section incorporating lines 39 and 40 read:

Of the 3595 studies screened, nine met the eligibility criteria. All studies were published after 2012, highlighting the recency of research in this area. Many were Australian studies (44%), and participants were from various allied health professions. Of the included studies, four related to undergraduate programs, three to postgraduate programs and for and two this was not specified. Curricula were delivered through a combination of online and face-to-face delivery, with assessment tasks, where reported, comprising mainly multiple choice and written tests.

With the updated results included and re-wording for correctness this now reads:

Of the 4484 studies screened, eleven met the eligibility criteria. All studies were published after 2012, highlighting the recency of research in this area. The studies were conducted in four countries (Australia, United Sates of America, United Kingdom, Norway), and participants were from various allied health professions. Of the included studies, four related to undergraduate programs, four to postgraduate programs and for the remaining three, this was not specified. Curricula were delivered through a combination of online and face-to-face delivery, with assessment tasks, where reported, comprising mainly multiple choice and written tests.

6. PLOS authors have the option to publish the peer review history of their article ([what does this mean?](https://protect-au.mimecast.com/s/xv5qC71RLWTPmMZnhA3ZyW?domain=journals.plos.org)). If published, this will include your full peer review and any attached files.


If you choose “no”, your identity will remain anonymous but your review may still be made public.


**Do you want your identity to be public for this peer review?** For information about this choice, including consent withdrawal, please see our [Privacy Policy](https://protect-au.mimecast.com/s/DwcHC81V6BTJj3YBS9Szvj?domain=plos.org).

Reviewer #1: **Yes:** Suptendra Nath Sarbadhikari

Reviewer #2: **Yes:** Dr Gayatri Ravulaparthi

**Additional changes to the manuscript**

In line with the updating of the review, with the search duration expanded to April 30^th^ 2021, there were two additional studies included in the review. This has resulted in minor changes eg reference numbers have been changed in response to these additional studies and data relating to these two studies have included in Tables. There have also been appropriate changes throughout in the Abstract, body of the Methods, Results, and Discussion sections (red track changes), to account for the additional studies and to consider the changes in this topic area in the period since the original manuscript was submitted.

**Authors’comment**

Thank you to the reviewers for your contribution to this review process.
